# Global profiling of ribosomal protein acetylation reveals essentiality of acetylation homeostasis in maintaining ribosome assembly and function

**DOI:** 10.1093/nar/gkad768

**Published:** 2023-09-23

**Authors:** Jinjing Ni, Shuxian Li, Yanan Lai, Zuoqiang Wang, Danni Wang, Yongcong Tan, Yongqiang Fan, Jie Lu, Yu-Feng Yao

**Affiliations:** Laboratory of Bacterial Pathogenesis, Shanghai Institute of Immunology, Shanghai Jiao Tong University School of Medicine, Shanghai 200025, China; Laboratory of Bacterial Pathogenesis, Shanghai Institute of Immunology, Shanghai Jiao Tong University School of Medicine, Shanghai 200025, China; Laboratory of Bacterial Pathogenesis, Shanghai Institute of Immunology, Shanghai Jiao Tong University School of Medicine, Shanghai 200025, China; Laboratory of Bacterial Pathogenesis, Shanghai Institute of Immunology, Shanghai Jiao Tong University School of Medicine, Shanghai 200025, China; Laboratory of Bacterial Pathogenesis, Shanghai Institute of Immunology, Shanghai Jiao Tong University School of Medicine, Shanghai 200025, China; Laboratory of Bacterial Pathogenesis, Shanghai Institute of Immunology, Shanghai Jiao Tong University School of Medicine, Shanghai 200025, China; College of Life and Health Sciences, Northeastern University, Shenyang 110819, China; Department of Infectious Diseases, Ruijin Hospital, Shanghai Jiao Tong University School of Medicine, Shanghai 200025, China; Laboratory of Bacterial Pathogenesis, Shanghai Institute of Immunology, Shanghai Jiao Tong University School of Medicine, Shanghai 200025, China; Department of Infectious Diseases, Ruijin Hospital, Shanghai Jiao Tong University School of Medicine, Shanghai 200025, China; State Key Laboratory of Microbial Metabolism, and School of Life Sciences and Biotechnology, Shanghai Jiao Tong University, Shanghai 200240, China; Shanghai Key Laboratory of Emergency Prevention, Diagnosis and Treatment of Respiratory Infectious Diseases, Shanghai 200025, China

## Abstract

Acetylation is a global post-translational modification that regulates various cellular processes. Bacterial acetylomic studies have revealed extensive acetylation of ribosomal proteins. However, the role of acetylation in regulating ribosome function remains poorly understood. In this study, we systematically profiled ribosomal protein acetylation and identified a total of 289 acetylated lysine residues in 52 ribosomal proteins (r-proteins) from *Salmonella* Typhimurium. The majority of acetylated lysine residues of r-proteins were found to be regulated by both acetyltransferase Pat and metabolic intermediate acetyl phosphate. Our results show that acetylation plays a critical role in the assembly of the mature 70S ribosome complex by modulating r-proteins binding to rRNA. Moreover, appropriate acetylation is important for the interactions between elongation factors and polysomes, as well as regulating ribosome translation efficiency and fidelity. Dysregulation of acetylation could alter bacterial sensitivity to ribosome-targeting antibiotics. Collectively, our data suggest that the acetylation homeostasis of ribosomes is crucial for their assembly and function. Furthermore, this mechanism may represent a universal response to environmental signals across different cell types.

## Graphical Abstract

**Graphical Abstract:**
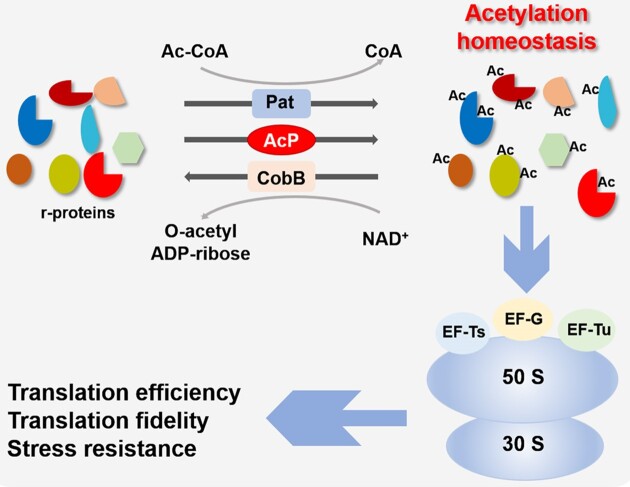

## INTRODUCTION

Ribosomes are essential cellular machines that translate mRNA into proteins. Despite differences in their composition, the basic structure of ribosomes is highly conserved in all cellular organisms (1). They are composed of two subunits, each made up of ribosomal RNAs (rRNAs) and ribosomal proteins (r-proteins). In bacteria, mature ribosomes consist of a 30S small subunit and a 50S large subunit, which contain 16S, 23S, and 5S rRNAs and 54 r-proteins (2). The assembly of ribosome involves ordered and correct association of r-proteins on rRNAs. The entire process is facilitated by the chaperoning activity of ribosome assembly factors, rRNAs and r-protein modification enzymes. The basic set of processes required to assemble a ribosome is detailed by biochemical approaches, mass spectrometry, computational methods, and others in recent years (3).

Ribosomes are involved in a highly dynamic and complex process of protein synthesis, which includes several steps: initiation, elongation, termination and recycling. During each of these phases, ribosomes form transient complexes with various translation factors that facilitate protein synthesis. For example, a codon exposed in the A site is recognized by aa-tRNAs, which are delivered to the ribosome in a ternary complex with EF-Tu and GTP. The initial recruitment of the EF-Tu/GTP/aa-tRNA complex occurs through the interactions with the L12 stalk of the ribosome (4,5). During elongation, the ribosome moves along the mRNA, adding amino acids to the growing polypeptide chain in response to each codon. The fidelity of protein synthesis is regulated by several mechanisms, including post-transcriptional modification of r-proteins, rRNA, and tRNA (6–9). However, the regulation of the efficiency and fidelity of protein synthesis is not completely understood.

Ribosomes are a common target for antibiotics, with many clinically important antibiotics, such as aminoglycosides, chloramphenicols, tetracyclines, and macrolides, targeting the elongation cycle. Chloramphenicol, for example, binds to the peptidyl-transferase center (PTC) on the bacterial 50S subunit, overlapping with the aminoacyl moiety of the A-site tRNA, thereby inhibiting peptide-bond formation by blocking aa-tRNA binding at the A-site. Resistance to ribosome-targeting antibiotics can occur through various mechanisms, including modification of the antibiotic targets, such as rRNAs or r-proteins. For example, mono-methylation of the C8 atom of the 23S rRNA nucleotide A2503 in the PTC can confer resistance to chloramphenicol (10). Modification of chloramphenicol to a phosphorylated derivative mediated by chloramphenicol phosphotransferase can also increase bacterial resistance (11). Overall, studies of ribosomes have provided valuable insights into the mechanisms of protein synthesis and the regulation of translation. Continued research on ribosomes will undoubtedly lead to the development of new antibiotics and further our understanding of the molecular basis of translation.

Acetylation is a post-translational modification (PTM) that plays a crucial role in regulating cellular processes in both prokaryotes and eukaryotes (12,13). This modification can occur on N^α^-amino groups (N termini of proteins) or N^ϵ^-amino groups of lysine residues. N^α^-acetylation is an irreversible modification that signals for protein degradation. For example, r-protein L7 is the N-terminally acetylated form of L12 (14,15). N^ϵ^-acetylation of lysine residues is reversible and some of them can be enzymatically reversed by deacetylases (16). N^ϵ^-acetylation can alter the biological activity of the protein, such as in bacterial metabolism, DNA replication, stress response and pathogenicity (17–20). Two distinct mechanisms have been identified in bacteria to regulate protein acetylation. The Gcn5-like acetyltransferase Pat/YfiQ can transfer the acetyl group from acetyl-CoA (Ac-CoA) to a deprotonated lysine residue on the target protein. The other mechanism is non-enzymatic, in which acetyl phosphate (AcP) serves as the acetyl donor to a deprotonated lysine. The synthesis of intracellular AcP in bacteria depends on phosphotransacetylase (Pta) and acetate kinase (AckA). Pta converts Ac-CoA and inorganic phosphate to AcP and free CoA, and then AckA converts AcP and ADP to acetate and ATP. Deletion of *ackA* increases the intracellular AcP level, while the double deletion of *ackA* and *pta* decreases the intracellular AcP concentration. CobB, the bacterial homolog of Sirt5, can remove acetyl groups from acetylated lysine residues (Kac) in an NAD^+^-dependent manner (21–23).

The first observation of r-protein acetylation was conducted by Liew and Gornall (24). Currently, the use of high-specificity antibodies to Kac sites, in combination with high-resolution mass spectrometry allows for the enrichment and identification of more acetylated peptides. For example, Choudhary and Weinert provide accurate and validated measurements of acetylation stoichiometry at 6829 sites on 2535 proteins in human cervical cancer (HeLa) cells (25). In-depth analyses of the bacterial protein acetylome datasets reveal that many r-proteins are acetylated, suggesting that acetylation may be involved in a feedback regulation of protein synthesis (26–29). Although studies have shown that acetylation affects the function of the translation machinery (30,31), the role of protein acetylation in regulating ribosome functions remains to be elucidated.

In this study, we analyzed the acetylation of r-proteins of *Salmonella enterica* serovar Typhimurium (*S*. Typhimurium) by mass spectrometry and found that the acetylation of r-proteins is regulated by both Pat and AcP. Acetylation is involved in regulating ribosome assembly, translation efficiency and fidelity, and sensitivity to ribosome-targeting antibiotics. Our data suggest that the acetylation homeostasis of r-proteins is critical to ribosome functions.

## MATERIALS AND METHODS

### Strains and media

Bacterial strains used in this study are listed in Supplementary Table S1. *S*. Typhimurium strain 14028S was purchased from ATCC and used as the wild-type *Salmonella* strain. All mutants derived from strain 14028S were constructed by one-step λ-Red recombinase system (32). All constructs were verified by PCR and sequencing, and PCR primers are listed in Supplementary Table S2. *S*. Typhimurium strains were grown in lysogeny broth (LB) with 100 μg/ml of ampicillin, 50 μg/ml of spectinomycin, 17 μg/ml of chloramphenicol or 50 μg/ml of kanamycin in media for bacterial selection.

### Plasmid construction and proteins purification

For overexpression of proteins in *S*. Typhimurium, genes encoding r-proteins (*rplL, rplX*, *rplB* and *rpS4*) and *tufA, tsf, fusA* were PCR amplified from the genomic DNA and cloned into the *Bam*HI and *Xho*I sites of the pSUMO3 and the pQE80 with 6 × His tag inserted at the C termini respectively. *ramA was* cloned into the *Eco*RI and *Nco*I sites of the expression plasmid pACYC184 with 3 × Flag tag inserted at the C terminus. *E. coli* strain BL21 harboring plasmids was grown at 37°C in LB medium. At OD_600_ = 0.6, 0.1 mM isopropyl β-D-thiogalactoside (IPTG) was added and induced at 30°C for 4 h. The harvested cells were resuspended in cold buffer A (50 mM Tris–HCl, pH 7.5, 500 mM NaCl, 20 mM imidazole, 10% glycerol), then cell suspension was lysed by pressure cell disrupters. The supernatant was collected and loaded onto a Ni-NTA column (GE), washed with buffer A, and finally eluted with buffer B (50 mM Tris–HCl, pH 7.5, 500 mM NaCl, 300 mM imidazole, 10% glycerol). Protein concentration was determined using the Bradford reagent with bovine serum albumin (BSA) as a standard (33).

### Antibodies

The following antibodies were used: anti-His peptide monoclonal antibody (TianGen), anti-Flag peptide monoclonal antibody (Sigma), anti-DnaK monoclonal antibody (Abcam), anti-mCherry mouse monoclonal antibody (Novus), and anti-EF-Tu monoclonal antibody (Hycult). Anti-L2 and -S4 polyclonal antibodies were prepared as follows: the purified 6 × His-tagged L2 and S4 were used as the antigens to immunize New Zealand rabbits three times to raise polyclonal antibodies. Anti-Kac polyclonal antibody was a gift from Dr. Shimin Zhao at Fudan University (22).

### Ribosome extraction and sucrose sedimentation

Ribosome isolation was adapted from previous methods (34). Briefly, bacteria were resuspended by buffer A (50 mM Tris–HCl at pH 7.5, 10 mM MgCl_2_, 100 mM NH_4_Cl, 0.5 mM EDTA and 6 mM 2-mercaptoethanol) with the addition of Complete Mini Protease Inhibitor cocktail (KangWei) and lysed by French press, and the supernatant was collected for filtration and sterilization with 0.22 μm filter membrane. The lysate was layered over a 36% sucrose cushion composed of buffer B (50 mM Tris–HCl at pH 7.5, 10 mM MgCl_2_, 500 mM NH_4_Cl, 0.5 mM EDTA and 6 mM 2-mercaptoethanol) and spun centrifuge at 120000 g for 16 h in a Beckman ultracentrifugation 41Ti rotor at 4°C. The ribosome pellets were washed once with buffer C (50 mM Tris–HCl at pH 7.5, 10 mM MgCl_2_, 100 mM NH_4_Cl and 6 mM 2-mercaptoethanol) and then resuspended in the same buffer by gentle rocking at 4°C. Purified ribosomes were profiled in 15–50% (w/v) sucrose gradients prepared in buffer C with 10 mM MgCl_2_ (associative conditions) or in 10–30% (w/v) sucrose gradients prepared in buffer C with 10 μM MgCl_2_ (dissociative conditions). Samples were centrifuged in a Beckman ultracentrifugation SW41 rotor for 16 h at 71 000 g at 4°C and analyzed by UV using the gradient fractionator system (Biocomp).

### Identification of acetylation by label free-mass spectrometry

The wild-type *S*. Typhimurium and mutant strains were grown in LB medium to log or stationary phase. R-proteins were collected by sucrose gradient centrifugation and digested with trypsin at 37°C for 18 h, then desalted on C18 column (Waters Wat051910). The acetylated peptides were enriched by anti-Kac antibody beads (PTMScan acetyl lysine motif (Kac) Kit, Cell Signal Technology) and then taken for LC–MS/MS analysis (Thermo Fisher Scientific). Mass spectrometric data were analyzed using the Maxquant software (version no. 1.3.0.5) for database search. To quantify the acetylation levels of r-proteins, the ratios of average area (representing peptide intensity) of acetylated peptides to the average area of total lysine-containing peptides were calculated.

### *In vitro* (de)acetylation assays

All *in vitro* (de)acetylation assays were performed as described (19,20). For Pat acetylation assay, L2 was incubated at 37°C for 2 h in the presence or absence of Pat as well as Ac-CoA. For CobB deacetylation assay, L2 protein was incubated at 37°C for 2 h in the presence or absence of CobB as well as NAD^+^. For AcP acetylation assay, L2 and L24 were incubated at 37°C for 2 h in the presence or absence of AcP at indicated concentrations.

### Western blot

Briefly, protein samples were separated by SDS-PAGE electrophoresis and transferred to PVDF membranes. For acetylation western blot, blocking buffer (50 mM Tris–HCl at pH 7.5, 100 mM NaCl, 10% (V/V) Tween-20 and 1% peptone (Amresco) was used for blocking. Non-fat milk buffer (50 mM Tris–HCl at pH 7.5, 150 mM NaCl, 0.5% (V/V) Tween-20 and 5% non-fat milk) was used for western blot. Horseradish peroxidase (HRP)-conjugated goat anti-rabbit or anti-mouse IgG was used as the secondary antibodies and incubated at room temperature for about 1 h. Blots were scanned with ECL Chemi System (Tanon) and relative intensity value was quantified by Image J.

### RNA extraction and quantitative real time- PCR assay

The wild-type *S*. Typhimurium and mutant strains were grown in LB medium overnight, diluted 1:100 to fresh LB medium and cultured at 37°C with vigorous shaking till OD_600_ = 1.0. Bacteria were collected and lysed with TRIZOL (Thermo Fisher Scientific) and extracted by RNAprep Pure Kit (Tiangen). Contaminated genomic DNA was removed by RNase-free DNase I (Thermo Fisher Scientific). For extraction of mRNA/rRNA binding to ribosomes, minor modifications were adapted from previous methods (35). Briefly, polysomes were purified by sucrose gradient centrifugation, then RNA was extracted with TRIZOL.

RNA samples were reversed transcribed with the random hexamers using Hifair™ II 1st Strand cDNA Synthesis Super Mix (Yesean). Quantitative reverse transcription PCR (qPCR) analysis was performed using SYBR Premix Ex Taq II (TaKaRa) in the QuantStudio3 fast real-time PCR system (Thermo Fisher Scientific). The 16S rRNA was used as a control and relative gene expression was calculated by 2^−ΔΔCT^ method.

### Bacterial spot plating assay

*S*. Typhimurium cells were grown in LB medium overnight and diluted 1:100 to fresh LB medium and cultured at 37°C with vigorous shaking. Bacteria were collected at OD_600_ = 1.0 and serially 10-fold diluted, and then spotted (2 μl of dilutions) on LB agar plates. Plates were photographed after 14 h of incubation at 37°C or 16°C.

### *In vitro* transcription assay

*In vitro* transcription assay was performed as described according to the operation of *In vitro* Transcription Kit (Thermo Fisher Scientific) (36). Briefly, linearized pCW1 plasmid by *Bspt*I (Thermo Fisher Scientific) was co-incubated with T7 RNA polymerase, 10x reaction buffer, ATP solution, CTP solution, GTP solution and UTP solution at 37°C for 1 h. The residual DNA was digested by DNase at 37°C for 1 h. and RNA was quantified on Nanodrop.

### Electrophoretic mobility shift assay (EMSA)

EMSA was performed using the purified L24 or its variants and 23S rRNA transcribed from pCW1 *in vitro*. 6 μg RNA was mixed with 3 μg proteins and incubated at 4°C for 1 h. The samples were analyzed by 1% agarose gel electrophoresis (150 V for 45 min). The gels were subjected to DNA dye staining for 5 min and photographed by using a gel imaging system (Tanon). The assay was repeated at least three times, and a representative result was shown.

### Translation fidelity determination

Translation fidelity was determined as described previously (37). pZS-mCherry-TGA-YFP plasmid was transformed into *S*. Typhimurium and mutant strains and cultured at 37°C for 24 h. The cells were lysed by sonication and whole protein samples were analyzed by western blot with anti-mCherry. The signals were qualified by Image J and the error rates were calculated as the percentage of mCherry-YFP fusion protein. The multicolor flow cytometry system (Beckman) was used to detect the mCherry-YFP fusion proteins. A total of 500 000 cells were harvested and placed into a 5 ml round bottom polystyrene tube (Falcon) and followed by data acquisition performed by mCherry and YFP signals. The assay was repeated at least three times, and a representative result was shown.

### L-azidohomoalanine (AHA) labeling assay

Labeling of nascent protein synthesis was performed using Click-iT Protein Reaction Buffer Kit (Thermo Fisher Scientific) according to the manufacturer's instructions. *Salmonella* strains were grown overnight in M9CA. Overnight cultures were washed three times in M9-minimal medium containing an amino acids mixture lacking methionine (1.6 mM of alanine, glycine, leucine, glutamate and serine, 1.2 mM glutamine and isoleucine, 0.8 mM arginine, asparagine, aspartate, lysine, phenylalanine, proline, threonine and valine, 0.4 mM histidine and tyrosine, and 0.2 mM cysteine and tryptophan). After 4 h growth, cultures were labeled with 40 μM of AHA for 30 min and lastly bacterial cultures were treated with 100 μg/ml of chloramphenicol to end nascent translation. Cell pellets were resuspended in a lysis buffer (50 mM Tris–HCl at pH 8.0, 0.5% sodium dodecyl sulfate (SDS) and 1× protease inhibitor cocktail). Cells were lysed by sonication and insoluble debris was removed by centrifugation. Covalent attachment of fluorescent tetramethylrhodamine (TAMRA)-alkyne (Thermo Fisher Scientific) to AHA containing proteins was carried out using Click-iT Protein Reaction Buffer Kit (Thermo Fisher Scientific). Proteins samples were separated by SDS-PAGE and fluorescent signals in gels were measured in a Gel Doc XR+ device (Bio-Rad). The relative rate of protein synthesis was estimated as the fluorescence signal normalized by the protein content of the sample by ImageJ.

### Thin-film interferometry (TFI) technology

A TFI assay was established on the GatorPrime label-free Analyzer instrument (GatorBio) and the binding ability of EF-Tu to L7/L12 or mutants was analyzed. Biotin was incubated with 40 μM of EF-Tu for 1 h at room temperature and purified by a PD-10 column. Each L7/L12 and mutant proteins sample was prepared in a serial dilution (12.5, 6.25, 3.125, 1.563, 0.781 and 0.391 μM) in binding buffer at pH 7.4 for analysis. Following loading, biotin-sensors were re-equilibrated for 10 min in binding buffer, then dipped into wells containing binding buffer for 30 s to establish a baseline. Biotin-sensors were dipped into wells containing r-proteins to monitor association signal and returned to wells containing binding buffer to monitor dissociation. Initial data processing was performed in Octet Data Analysis 10.0 or Gator 1.7 software.

## RESULTS

### Lysine acetylation is widely present in r-proteins and regulated by both non-enzymatic and enzymatic mechanisms

**Figure 1.**
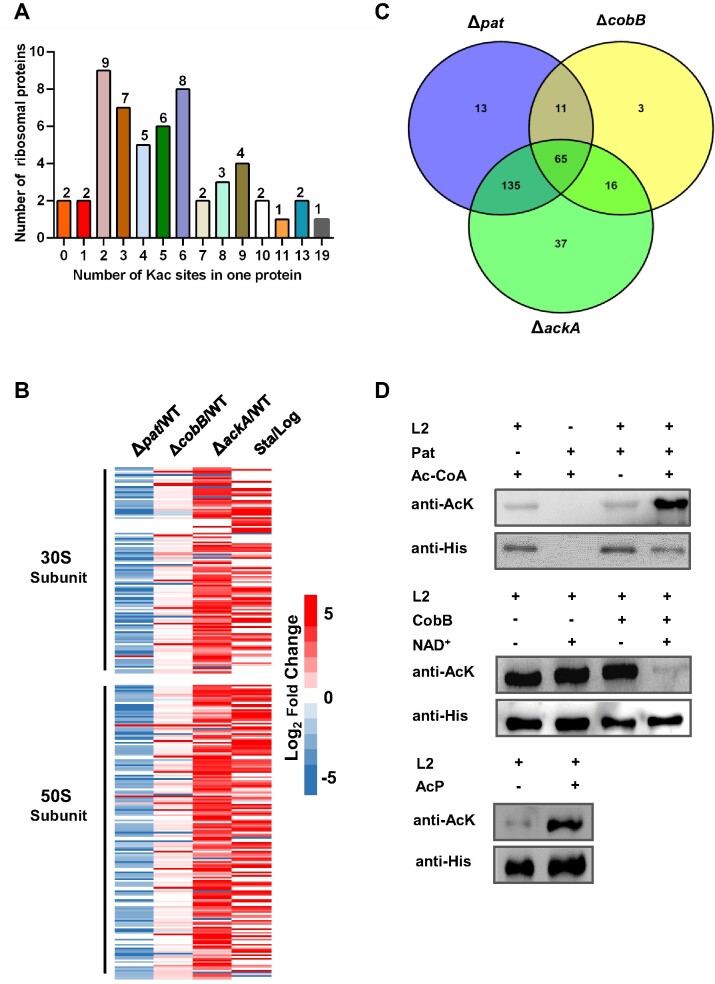
Lysine acetylation is widely present in r-proteins. (**A**) Analysis of Kac sites in r-proteins. The numbers of r-proteins containing 0–19 Kac sites were labelled above the column. (**B**) Heatmap highlighting ribosomes subunit clusters. A cluster analysis was performed using the Log_2_ Fold Change values of the 289 Kac sites of Δ*pat*, Δ*cobB* and Δ*ackA* strains compared to WT in log phase or WT in stationary phase compare to WT in log phase. (**C**) The Venn diagrams identified the numbers of Kac sites regulated by Pat, CobB and AcP. (**D**) *In vitro* (de)acetylation assay of ribosome large subunit protein L2. The His-tagged L2 (0.1 μg/μl) was purified by Ni-NTA column and co-incubated with Pat (10 μg) in the presence of Ac-CoA (0.2 mmol/L), CobB (0.1 μg/μl) in the presence of NAD^+^ (1 mmol/L), and AcP (5 μmol/L). The acetylation levels were determined by anti-Kac antibody. Western blots are representative from at least three independent replicates.

Many bacterial acetylome studies have identified acetylated r-proteins. However, these studies usually used whole-cell lysates as starting material, which may lead to the underrepresentation of r-proteins and failure to identify acetylated r-proteins (26). To address this issue, we employed a systematic approach to identify lysine acetylation of r-proteins in strains with different acetylation levels. We used wild-type (WT) strain, hypoacetylation strain Δ*pat*, AcP-accumulated strain Δ*ackA* and deacetylase-knockout strain Δ*cobB*. Briefly, we collected ribosomes from WT, Δ*pat*, Δ*cobB* or Δ*ackA* cells in exponential phase and WT cells in stationary phase using sucrose gradient centrifugation, enriched acetylated peptides with specific antibodies, then detected lysine acetylation by label-free high-resolution LC–MS/MS (Supplementary Figure S1A). The results showed that a total of 289 Kac sites were identified in 52 of 54 r-proteins (Supplementary Table S3). Most r-proteins contained 2 or more Kac sites except for L35 and L36 (Figure 1A). Small subunit protein S1, the largest r-protein, contained 19 Kac sites.

We compare the frequency of lysine residues in Salmonella r-proteins and whole-genome proteins and find that lysine residues are highly enriched in r-proteins. The percentage of lysine residues in r-proteins and across the whole genome of *Salmonella* was 9.6% and 4.4% on average, respectively (Supplementary Figure S1B). Interestingly, the number of Kac was positively correlated with the total number of lysine residues among r-proteins (Supplementary Figure S1C).

We compared quantitatively the lysine acetylation levels of r-proteins in three acetylation-modulating mutants with the WT strain. The results showed that the acetylation level of 224 lysine sites (77.5%) decreased in Δ*pat*. Deletion of *ackA* can lead to the accumulation of intracellular AcP, while the acetylation level of 253 lysine sites (87.5%) increased in Δ*ackA* correspondingly. In contrast, deletion of *cobB* did not greatly alter the acetylation pattern of r-proteins, except for an increase in the acetylation levels of 95 lysine sites. Additionally, we found that the acetylation levels of 182 lysine sites (63.0%) in stationary phase were higher than those in the exponential phase (Figure 1B). Interestingly, we analyzed the Kac sites of r-proteins in the *pat* and *ackA* mutants, and found that 200 Kac sites were regulated by both Pat and AcP, suggesting that non-enzymatic and enzymatic mechanisms could jointly modulate acetylation of r-proteins (Figure 1C).

To verify the acetylome results, we purified the large subunit r-protein L2 (simplified as L2) and performed (de)acetylation assay *in vitro* (Figure 1D). The results of western blot showed that the acetylation level of L2 increased significantly after co-incubation with Pat and Ac-CoA. CobB could deacetylate L2 in the presence of NAD^+^. Additionally, co-incubation with AcP significantly increased L2 acetylation level. These results indicated that r-proteins were widely acetylated, and most sites were jointly acetylated by Pat and AcP.

### Acetylation is involved in ribosome assembly

**Figure 2.**
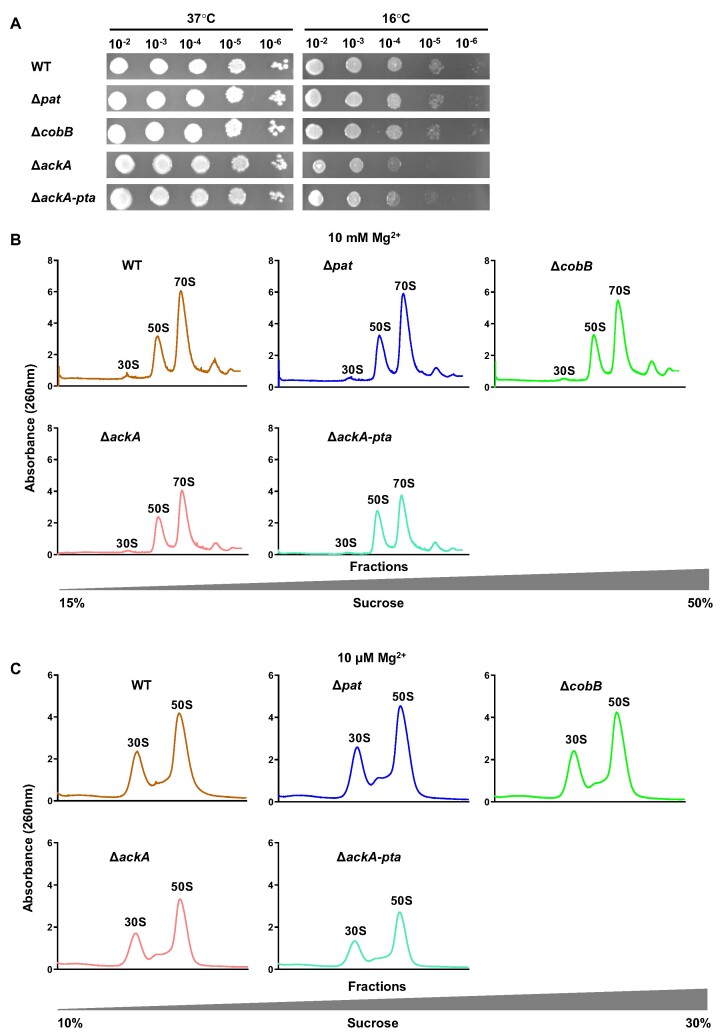
Acetylation is involved in ribosome assembly. (**A**) Spot plating assay of WT, Δ*pat*, Δ*cobB*, Δ*ackA* and Δ*ackA-pta* strains. The overnight cultured bacteria were 1:100 diluted in fresh LB medium. After incubation at 37°C for about 2 h, the bacteria were collected at an OD_600_ of 1.0. The bacterial cells were serially 10-fold diluted from left to right and then spotted on LB agar plates. Plates were photographed after 14 h of incubation at 37°C or 16°C. (**B**) Polysome profiling of WT and mutant strains in associative conditions. Ribosomes from log phase bacteria including of WT, Δ*pat*, Δ*cobB*, Δ*ackA* and Δ*ackA-pta* strains were isolated by ultracentrifugation in 15%-50% (w/v) sucrose gradients solution with 10 mM MgCl_2_. The amount of ribosome was monitored by optical absorbance at 254 nm. (**C**) Polysome profiling of WT and mutant strains in dissociative conditions. Ribosomes from log phase bacteria including of WT, Δ*pat*, Δ*cobB*, Δ*ackA* and Δ*ackA-pta* strains were isolated by ultracentrifugation in 10–30% (w/v) sucrose gradients solution with 10 μM MgCl_2_. The amount of ribosome was monitored by optical absorbance at 254 nm.

In order to explore the role of protein acetylation in regulating ribosome function, we compared the competency of ribosome assembly among the above-mentioned strains. Growth defect at low temperature usually indicates impaired ribosome assembly (38). We cultured these strains (WT, Δ*pat*, Δ*cobB*, Δ*ackA* and Δ*ackA-pta*) at 16°C overnight and the spot plating results showed that deletion of *ackA* or double deletion of *ackA* and *pta* caused significant growth defects, while Δ*pat* and Δ*cobB* had slight growth defects at low temperature (Figure 2A), suggesting that acetylation may be involved in the ribosome biogenesis process.

To consolidate these results, we performed polysome profiling using 10 mM Mg^2+^ buffer and found that the abundance of mature 70S ribosomes in Δ*ackA* and Δ*ackA-pta* was reduced by about 25% compared with that of WT in the exponential phase (Figure 2B). This suggests that dysregulation of acetylation leads to a reduction of 70S ribosomes in the cell. To distinguish between the possibilities of whether the reduction in 70S ribosomes was due to the defect of subunits formation or the inability of large and small subunits to assemble into a mature ribosome (38), we further profiled polysomes under dissociative conditions (10 μM Mg^2+^) to guarantee that all ribosomal subunits would be in their free state and cannot combine into the mature 70S ribosomes (39). We found that the amount of 30S and 50S subunits in Δ*ackA* and Δ*ackA-pta* strains was significantly reduced in dissociative conditions (Figure 2C).

### Acetylation regulates translation factors binding to ribosome

During protein translation, single ribosome displays relatively low translation efficiency. Therefore, multiple ribosomes can associate with a single mRNA to form a polysome, which enables the synthesis of multiple peptide chains simultaneously and improves translation efficiency significantly (40). In addition to the ribosome itself, other components, such as elongation factors and GTP, are required to facilitate protein synthesis (41). To investigate whether acetylation affects polysome profile, we collected polysomes from the WT strain and the hyperacetylation strain (Δ*ackA*) and subjected them to quantitative analysis of polysome composition and ribosome-associated proteins using label-free mass spectrometry. Our data showed that polysomes from both WT and Δ*ackA* strains had similar r-protein compositions (Supplementary Table S4), suggesting that acetylation does not affect major ribosome components. Interestingly, Clusters of Orthologous Genes analysis revealed a decrease in polysome-binding proteins clustered in transcription and translation pathways in Δ*ackA* (Supplementary Figure S2A). Specifically, the amount of several translation elongation factors including EF-Tu, EF-Ts and EF-G was reduced in Δ*ackA* compared to WT (Supplementary Figure S2B).

**Figure 3.**
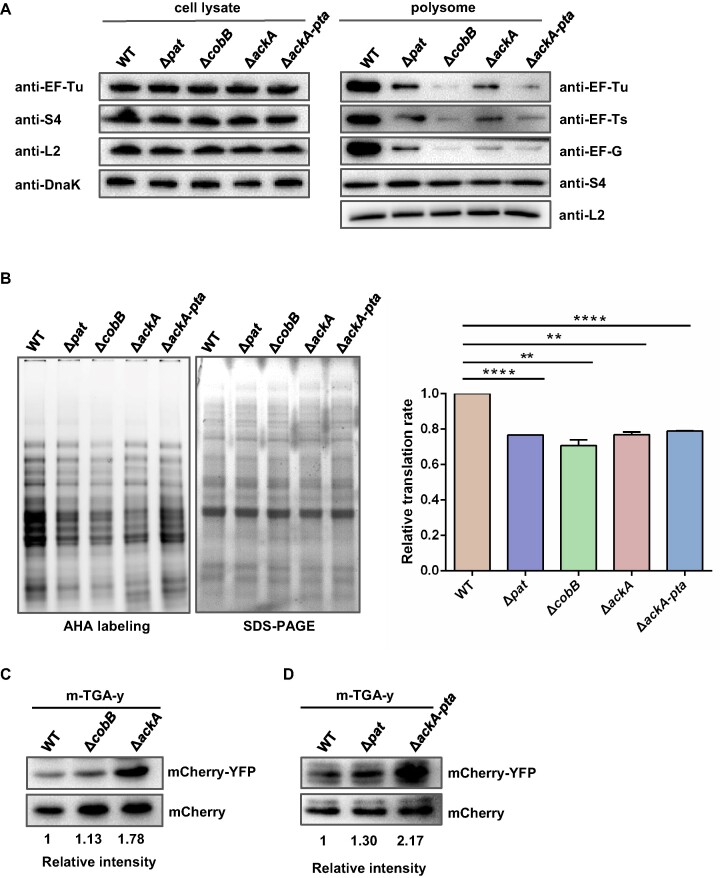
Acetylation alters ribosome association with translation factors, translation efficiency and fidelity. (**A**) Amount of translation elongation factors in cell lysates and polysomes of WT, Δ*pat*, Δ*cobB*, Δ*ackA* and Δ*ackA-pta* strains. Bacteria were cultured in LB medium to log phase, and the cell lysates and polysomes were collected. The levels of r-proteins were determined by anti-L2 and anti-S4 antibodies. The levels of elongation factors were detected by anti-EF-TU, anti-EF-Ts and anti-EF-G antibodies, and DnaK was used as a loading control. Western blots are representative from at least three independent replicates. (**B**) Translation efficiency analysis of WT, Δ*pat*, Δ*cobB*, Δ*ackA*, and Δ*ackA-pta* strains. Bacteria were labeled with AHA, and the signal of tetramethylrhodamine (TAMRA) was detected by fluorescence imaging system. SDS-PAGE was used as protein loading control. Results are representative of three biological replicates. Two technical replicates are shown. Data are presented as mean values ± SD, unpaired Student's *t*-test. ** *P* < 0.01, ****P < 0.0001. (**C**) Translation fidelity analysis of hyperacetylation strains (Δ*cobB* and Δ*ackA*). Fidelity was represented by mCherry-YFP fusion protein level, and mCherry was used as a control. (**D**) Translation fidelity analysis of hypoacetylation strains (Δ*pat* and Δ*ackA-pta*). Fidelity was represented by mCherry-YFP fusion protein level, and mCherry was used as a control.

Given that deletion of *ackA* increases intracellular AcP and enhances ribosomal protein acetylation (Figure 1B), it seems that acetylation may regulate the binding of translation elongation factors to polysomes. To test this hypothesis, we isolated polysomes from strains including WT, Δ*pat*, Δ*cobB*, Δ*ackA* and Δ*ackA-pta* and examined the polysome-bound translation elongation factors by western blot (Figure 3A). Our results demonstrate that deletion of acetylation-related genes did not alter the expression level of ribosomal proteins or translation elongation factors. However, the amount of polysome-bound translation elongation factors, including EF-Tu, EF-Ts and EF-G, was significantly reduced in the mutant strains compared to WT regardless of polysome acetylation status.

### Acetylation of r-proteins alters ribosome translation efficiency and fidelity

The acetylation of r-proteins can affect ribosome assembly and the association of ribosome to translation elongation factors, which could ultimately affect the ribosome's translation function. To investigate this, we utilized AHA, a methionine analogue, to determine the translation efficiency of ribosome *in vivo* (42). AHA is incorporated into proteins during active protein synthesis and after a click reaction between an azide and an alkyne. The azide-containing proteins can be detected with an alkyne-tagged fluorescent dye, coupled with biochemistry techniques such as gel electrophoresis. We first conducted AHA labeling in WT, Δ*pat*, Δ*cobB*, Δ*ackA* and Δ*ackA-pta* strains. The AHA incorporation assay results showed that translation efficiencies in all mutant strains were lower than that in WT (Figure 3B). Deletion of either acetylation-modulating gene decreased the relative translation efficiencies by about 25%.

Ribosomes provide a series of mechanisms to ensure the fidelity of translation during the process (7,43,44). A reduction in protein synthesis fidelity would lead to an accompanying increase in mistranslation, which can be harmful to the cell. To investigate whether acetylation is involved in protein translation fidelity (45), we inserted an in-frame YFP tag immediately after the termination codon TGA of the mCherry gene. Normally, cells only synthesize mCherry protein, but stop codon readthrough can cause the synthesis of the mCherry-YFP fusion protein. We transformed the plasmid into WT, Δ*pat*, Δ*cobB*, Δ*ackA* and Δ*ackA-pta* strains individually. Firstly, we determined the mCherry-YFP fusion protein expression in the hyperacetylation level strains (Δ*cobB* and Δ*ackA*). The fusion protein in Δ*ackA* was upregulated by 1.78-fold compared to that in WT, while deletion of *cobB* did not alter the fusion protein level (Figure 3C). Then, we analyzed the fusion protein expression in the hypoacetylation level strains and found that the level of fusion protein in Δ*pat* and Δ*ackA-pta* was increased by 1.30- and 2.17-fold, respectively (Figure 3D). These results suggest that acetylation plays an important role in regulating ribosome translation fidelity in bacteria.

### Acetylation of L24 K33 and L7/L12 K65, K70 are involved in ribosome assembly

**Figure 4.**
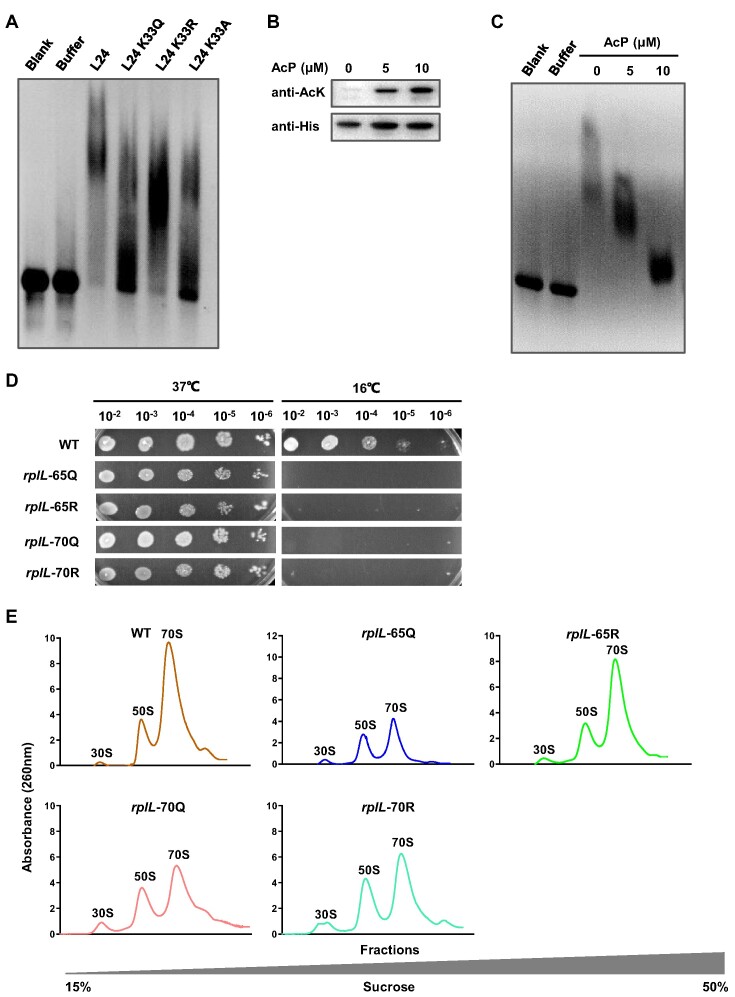
Acetylation of L24 K33 and L7/L12 K65, K70 are involved in ribosome assembly. (**A**) 23S rRNA binding activity of L24 and L24 K33Q, K33R, K33A by EMSA. The indicated amounts of L24 and variant proteins were incubated with 23S rRNA transcribed *in vitro* and followed by EMSA analysis. The blank and protein buffer were used as negative controls. (**B**) *In vitro* AcP modification assay of L24. L24 (3 μg) was purified and incubated with 0, 5, and 10 μM AcP at 37°C for 2 h. Acetylation of L24 was determined by western blot using anti-Kac antibody. (**C**) 23S rRNA binding activity of L24 modified AcP. L24 (3 μg) pretreated by indicated concentrations of AcP was applied to EMSA analysis. (**D**) Spot plating assay of WT, *rplL*-65Q, *rplL*-65R, *rplL*-70Q and *rplL*-70R strains. The overnight cultured bacteria were 1:100 diluted in fresh LB medium. After incubation at 37°C for about 2 h, the bacteria were collected at an OD_600_ of 1.0. The bacterial cells were serially 10-fold diluted from left to right and then spotted on LB agar plates. Plates were photographed after 14 h of incubation at 37°C or 16°C. (**E**) Polysome profiling of WT and mutant strains in associative conditions. Ribosomes from log phase bacteria including of WT, Δ*pat*, Δ*cobB*, Δ*ackA* and Δ*ackA-pta* strains were isolated by ultracentrifugation in 15%-50% (w/v) sucrose gradients solution with 10 mM MgCl_2_. The amount of ribosome was monitored by optical absorbance at 254 nm.

To further confirm the role of acetylation in ribosome assembly, we selected specific r-protein Kac sites to examine whether acetylation directly regulates ribosome assembly. During the ribosome assembly process, ribosomal proteins L4, L13, L20, L22, L24 and 23S rRNA are essential for the large subunit intermediate *in vivo* and conformational intermediate change *in vitro* (46,47). Our mass spectrometry data revealed that the acetylation level of L24 at lysine 33 (L24 K33) was upregulated in Δ*ackA* and stationary-phase WT cells (Supplementary Table S3). Analysis of the crystal structure of L24 and 23S rRNA indicated that K33 was close to C86 and A91 nucleotides of 23S rRNA (PDB number: 4V6G) (Supplementary Figure S3A). Therefore, we hypothesize that acetylation of K33 may be involved in L24’s interaction with 23S rRNA, considering the location and positive charge of this lysine residue. To test this hypothesis, we mutated L24 K33 to arginine (R), glutamine (Q) or alanine (A). The K-to-R substitution prevented acetylation but kept positive charge, mimicking the non-acetylated form, and the K-to-Q substitution mimicked the constitutively acetylated form through neutralization of the positive charge. We then expressed and purified WT L24 and its variants, including L24 K33R, L24 K33Q and L24 K33A (Supplementary Figure S3B). All of these r-proteins were incubated with 23S rRNA obtained from *in vitro* transcription assay (48). The gel mobility assay results showed that L24 K33Q, K33R and K33A had weaker binding to 23S rRNA compared to WT L24 (Figure 4A).

The mass spectrometry data showed that intracellular AcP was involved in acetylation of L24 (Supplementary Table S3), so we co-incubated L24 with AcP at different concentrations *in vitro*. The acetylation level of L2 increased significantly in a concentration-dependent manner after co-incubation with AcP (Figure 4B). We then mixed these AcP-pretreated L24 proteins with the 23S rRNA obtained from *in vitro* transcription assay and performed EMSA. The results showed that the binding abilities of L24 with 23S rRNA were negatively correlated with its acetylation levels (Figure 4C).

In bacterial ribosomes, four copies of L7/L12 are bound as two dimers via their N-terminal domains (NTD) to L10, and L10 is attached to the rRNA. Our MS data showed that lysine residues at positions 59, 65, 70, 81, 84, 95, 100 and 107 on large subunit protein L7/L12 (also known as RplL) could be acetylated by Pat or AcP (Supplementary Table S3). Among these residues, K65, K70, K81 and K84 in the L7/L12 are highly conserved in diverse bacteria (Supplementary Figure S4A). The deletion of *pat* reduced the acetylation levels of K65 and K70 by 6.7 and 8.3 times, respectively (Supplementary Figure S4B). Deletion of *ackA* increased the acetylation levels of K65 and K70 by 11.4 and 6.2 times, respectively. Acetylation of K84 was mainly regulated by Pat and did not change much along with growth phase, while K81 acetylation was down-regulated in the stationary phase (Supplementary Figure S4B). We then focused on K65 and K70 in the subsequent study. In order to investigate the role of L7/L12 protein acetylation in ribosome assembly, we constructed the corresponding strains of L7/L12 K65Q, L7/L12 K65R, L7/L12 K70Q and L7/L12 K70R by chromosome knock-in system (49).

The spot plating assay showed that all the mutant strains had significant growth defects compared with their parental strain (Figure 4D), suggesting that acetylation of K65 and K70 were essential in the ribosome biogenesis process. The results of polysome profiling showed that the mature 70S ribosomes abundance of L7/L12 K65Q and L7/L12 K70Q was reduced by about 60%-80% compared with that of WT in the log phase, L7/L12 K65R and L7/L12 K70R was reduced by about 50–60% (Figure 4E).

### Acetylation of L7/L12 K65 and K70 is involved in ribosome association to EF-Tu, translation efficiency and fidelity

We found that the levels of polysome-bound translation elongation factors EF-Tu, EF-Ts and EF-G decreased significantly in the mutant strains compared to the WT, regardless of the acetylation states of polysome (Figure 3A). In bacterial ribosomes, the C-terminal domain (CTD) of 50S subunit proteins L7/L12 is necessary for the recruitment of elongation factors EF-Tu and EF-G to the ribosome and for the stimulation of GTP hydrolysis by the ribosome-bound factors through stabilization of their active GTPase conformation (50). Crystal structures (EF-Tu PDB number: 1EFU, L7/L12 PDB number: 1CTF) reveal that lysine residues K65 and K70 located in the large subunit protein L7/L12 CTD have strong binding affinity with L145 and L148 of EF-Tu (Supplementary Figure S4C). Furthermore, our mass spectrometry data showed that K65 and K70 in L7/L12 could be acetylated by both Pat and AcP (Supplementary Table S3).

**Figure 5.**
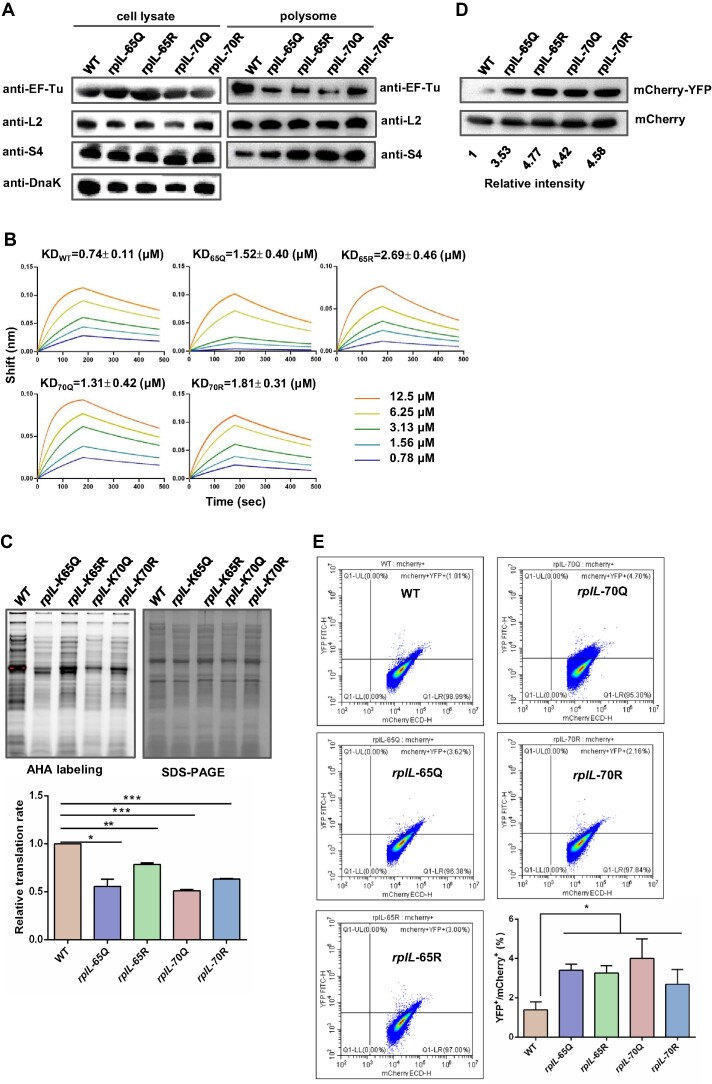
Acetylation of L7/L12 K65 and K70 is involved in ribosome association to EF-Tu, translation efficiency and fidelity. (**A**) Amount of r-proteins and EF-Tu in cell lysates and polysomes of WT, *rplL*-65Q, *rplL*-65R, *rplL*-70Q, and *rplL*-70R strains. Bacteria were cultured in LB medium to log phase, and the cell lysates and polysomes were collected. The levels of r-proteins were determined by anti-L2 and anti-S4 antibodies. The levels of elongation factors were detected by anti-EF-TU, and DnaK was used as a loading control. Western blots are representative from at least three independent replicates. (**B**) Analysis of EF-Tu binding to L7/L12, K65Q, K65R, K70Q, K70R by TFI. His-tagged EF-Tu and L7/L12, K65Q, K65R, K70Q, K70R were purified by Ni-NTA column. Biotin was incubated with 40 μM of EF-Tu for 1 h at room temperature, and followed by a PD-10 column purification. L7/L12 and its variant proteins were serially diluted (12.5, 6.25, 3.125, 1.563, 0.781 and 0.391 μM) in binding buffer for TFI analysis. Biotin-sensors were used to monitor association signal. The KD data represent the mean values and standard errors from three independent experiments. (**C**) Translation efficiency analysis of *rplL* mutant strains. Bacteria (WT, *rplL*-65Q, *rplL*-65R, *rplL*-70Q, and *rplL*-70R) were labeled with AHA, and the signal of TAMRA was detected by fluorescence imaging system. SDS-PAGE was used as protein loading control. Results are representative of three biological replicates. Two technical replicates are shown. Data are presented as mean values ± SD, unpaired Student's t-test. *P < 0.05, **P < 0.01, ***P < 0.0005. (**D**) Translation fidelity analysis of *rplL* mutant strains. Fidelity was represented by mCherry-YFP fusion protein level, and mCherry was used as a control. (**E**) Translation fidelity analysis by flow cytometry. Bacteria including WT, *rplL*-65Q, *rplL*-65R, *rplL*-70Q and *rplL*-70R were harvested and followed by data acquisition by mCherry and YFP signals. Results are representative of three biological replicates. Two technical replicates are shown. Data are presented as mean values ± SD, unpaired Student's *t*-test. *P < 0.05.

To further confirm the role of L7/L12 protein acetylation in elongation factors association, polysomes of WT and these *rplL*-65Q, *rplL*-65R, *rplL*-70Q and *rplL*-70R strains were isolated. Western blot results indicated that mutation of K65 or K70 in L7/L12 reduced the binding of translation elongation factors to polysomes regardless of whether the K to Q or K to R mutation occurred (Figure 5A).

Next, we purified L7/L12 and its variant proteins from *E. coli* and determined the binding affinities of these proteins to EF-Tu using TFI. The results showed that the equilibrium dissociation constant (KD) values between WT L7/L12 and EF-Tu were 0.74 ± 0.11 μM, while the KD values between 65Q, 65R, 70Q and 70R and EF-Tu were 1.52 ± 0.40, 2.69 ± 0.46, 1.31 ± 0.42 and 1.81 ± 0.31 μM, respectively (Figure 5B). The binding properties of the variant proteins to EF-Tu were significantly weaker than those of L7/L12. These results suggest that the acetylation state of r-proteins is crucial for regulating their binding with translation elongation factors.

We further used the r-protein (de)acetylation-mimic mutant strains to examine the role of acetylation in manipulating translation efficiency and fidelity. Since L7/L12 K65 and K70 are involved in elongation factors binding, we hypothesize that the acetylation state of K65 and K70 could alter the translation efficiency and fidelity of ribosomes. As expected, the AHA incorporation assay results showed that the relative translation efficiencies of L7/L12 K65Q and K70Q strains were 53.2% and 51.2% of WT level, respectively (Figure 5C). Interestingly, the relative translation efficiencies of L7/L12 K65R and K70R decreased by about 25%. Western blot results showed that the translation fidelity of all four mutants was lower than that of WT. Compared to WT, the levels of mCherry-YFP fusion proteins in K65Q, K65R, K70Q and K70R increased to 3.53-, 4.77-, 4.42- and 4.58-fold of WT level, respectively (Figure 5D). We further used flow cytometry to determine ribosome translation fidelity, the average percentage of YFP^+^ cells in the mCherry^+^ cells in WT is 1.20%, while the average percentages of YFP^+^/mCherry^+^ in these 4 mutants increased to 3.40%, 3.26%, 4.00% and 2.68%, respectively (Figure 5E).

### Ribosome acetylation is involved in bacterial stress susceptibility

**Figure 6.**
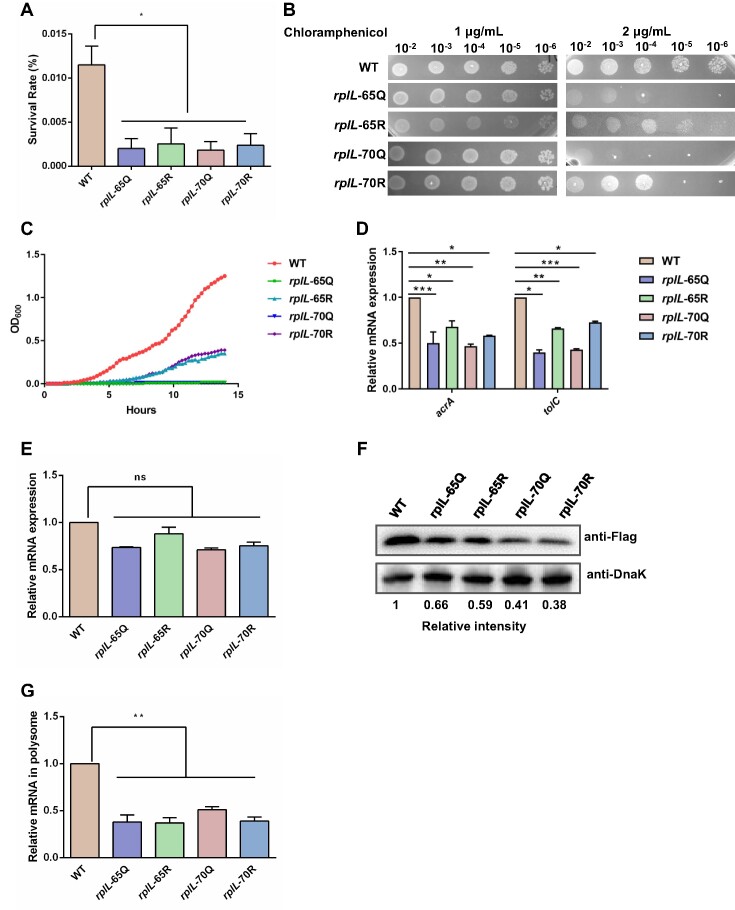
Ribosome acetylation is involved in bacterial stress susceptibility. (**A**) Survival rates of *rplL* mutant strains in bile salt. Bacteria (WT, *rplL*-65Q, *rplL*-65R, *rplL*-70Q, and *rplL*-70R) were cultured in the LB medium supplemented with 10% bile salt for 3 h. Survival rate is calculated as the ratio of the number of colonies obtained after and before bile salt treatment. Results are representative of three biological replicates. Two technical replicates are shown. Data are presented as mean values ± SD, unpaired Student's t-test. *P < 0.05. (**B**) Spot plating assay of *rplL* mutant strains in chloramphenicol medium. The overnight cultured bacteria including WT, *rplL*-65Q, *rplL*-65R, *rplL*-70Q and *rplL*-70R strains were 1:100 diluted in fresh LB medium. After incubation at 37°C for about 2 h, the bacteria were collected at an OD_600_ of 1.0. Then, bacteria were serially 10-fold diluted from left to right and then spotted on LB agar plates supplemented with 1 or 2 μg/ml chloramphenicol. (**C**) Growth curves of WT, *rplL*-65Q, *rplL*-65R, *rplL*-70Q, and *rplL*-70R strains in sub-MIC chloramphenicol broth. The overnight cultured bacteria were diluted to OD_600_= 0.04 in fresh LB supplemented with 1 μg/ml chloramphenicol and cultured at 37°C along with OD_600_ measurements. (**D)** qPCR analysis of *acrA* and *tolC* in WT, *rplL*-65Q, *rplL*-65R, *rplL*-70Q, and *rplL*-70R strains. All mRNA levels were normalized to the 16S rRNA level and expressed as the fold change compared to WT. Results are representative of three biological replicates. Two technical replicates are shown. Data are presented as mean values ± SD, unpaired Student's t-test. *P < 0.05, **P < 0.01, ***P < 0.0005. (**E**) qPCR analysis of *ramA* in WT, *rplL*-65Q, *rplL*-65R, *rplL*-70Q, and *rplL*-70R strains. All mRNA levels were normalized to the 16S rRNA level and expressed as the fold change compared to WT. Results are representative of three biological replicates. Two technical replicates are shown. Data are presented as mean values ± SD. (**F**) Expression of RamA in WT, *rplL*-65Q, *rplL*-65R, *rplL*-70Q, and *rplL*-70R strains. *ramA* was flag-tagged in chromosome using λ-red recombinase system. The levels of RamA were determined by anti-Flag antibody, and DnaK was used as a loading control. Western blots are representative from at least three independent replicates. (**G**) qPCR analysis of *ramA* in polysome of WT, *rplL*-65Q, *rplL*-65R, *rplL*-70Q and *rplL*-70R strains. All mRNA levels were normalized to the 16S rRNA level and expressed as the fold change compared to WT. Results are representative of three biological replicates. Two technical replicates are shown. Data are presented as mean values ± SD, unpaired Student's t-test. **P < 0.01.

Multiple studies have reported that protein mistranslation contributes to bacterial environmental adaptation (43,51–53). To investigate whether ribosome acetylation affects bacterial adaptation to environmental stresses, we determined the survival rates of five strains (WT, *rplL*-65Q, *rplL*-65R, *rplL*-70Q, *rplL*-70R) in LB medium with different additives. Our results showed that the survival rates of all mutant strains in LB medium supplemented with 10% bile salt decreased dramatically compared to the WT strain (Figure 6A). Similarly, all mutant strains were more sensitive to treatment with chloramphenicol at sub-minimal inhibitory concentration (MIC) (2 μg/ml) in agar medium blot assay (Figure 6B). Furthermore, all the mutants exhibited growth defects in LB medium supplemented with sub-MIC chloramphenicol compared to WT (Figure 6C). Interestingly, mutation of both K65 and K70 to R rendered higher resistance to chloramphenicol exposure than Q mutation (Figure 6B and C). This finding suggest that the acetylated status at K65 and K70 could attenuate bacterial resistance to antibiotic treatment.

AcrAB and TolC are responsible for the transport of bile acids and chloramphenicol towards the extracellular environment (54). Therefore, we assessed the transcription levels of *acrA* and *tolC* in the above 5 strains and observed that the expression levels of these two genes decreased by approximately two-fold in the mutants (Figure 6D). In *Salmonella*, the transcription of *acrAB* and *tolC* genes is mainly controlled by RamA (55). Subsequently, we evaluated the expression levels of *ramA* gene in these mutant strains and WT. The qPCR results demonstrated that the mRNA levels of *ramA* in the mutants were comparable to those in the WT (Figure 6E), but the protein levels of RamA in these mutants decreased dramatically (Figure 6F), suggesting that acetylation of r-proteins potentially regulates RamA at the post-transcriptional level. To validate this speculation, we isolated polysomes from WT, *rplL*-65Q, *rplL*-65R, *rplL*-70Q and *rplL*-70R cells and carried out RT-qPCR. The results showed that *ramA* mRNA was significantly less enriched in polysomes from these mutant strains, indicating that the translation efficiency of *ramA* was suppressed in the mutant strains (Figure 6G). These results indicate that acetylation of ribosomes potentially modulates protein translation and is likely involved in bacterial environmental adaptation.

## DISCUSSION

Ribosomes have long been considered essential structures that are consistent in composition within the same species. However, recent studies have demonstrated that r-proteins undergo various PTMs, including phosphorylation (56), methylation (57), ubiquitination (58) and acetylation (59). In this study, we systematically analyzed the ribosome acetylome in *Salmonella* and identified 289 Kac sites in 52 r-proteins. Our findings suggest that enzymatic and non-enzymatic lysine acetylation is a significant global PTM of r-proteins and critical to bacterial ribosomal assembly and function.

### The ubiquitous acetylation of r-proteins

The ribosome is a highly conserved translational machine in eukaryotes, archaea and prokaryotes, with most r-proteins shared among these three kingdoms. As RNA-binding proteins, r-proteins have a high content of basic amino acids including lysine residue, which is much more abundant than other amino acid residues (60). Lysine residue has a long side chain and can undergo a wide range of reversible PTMs, which can regulate enzyme activities, protein-protein interactions, protein-DNA interactions, protein stability, and protein cellular localization. Advances in mass spectrometry-based proteomics have revealed that, in addition to histones, lysine acetylation is a widespread PTM that occurs on a large number of proteins with diverse biological functions in various organisms (61,62). Bacterial acetylomes have been characterized in numerous species (17,21,26). Impressively, proteins involved in protein translation were found to be the second most acetylated proteins after those involved in central metabolism (26). However, due to limitations in protein isolation and mass spectrometry techniques, only a limited number of acetylated r-proteins have been identified so far. For example, in a previous study using *S*. Typhimurium cell lysates, we identified a total of 235 acetylated peptides that matched 191 proteins, with only 12 acetylated peptides from 12 r-proteins (22). Encouragingly, our current study identified 289 Kac sites in 52 r-proteins using purified ribosomes from cells in different states. Our results tie well with recent studies: Feid *et al.* analysed from published proteomics data set of 48 bacteria and found that r-proteins are highly acetylated and the Kac sites are conserved in diverse species of bacteria (63,64). In rice, HDA714 is identified as a major deacetylase of many r-proteins and translation factors that are extensively acetylated (59). Given the high abundance of lysine residues in ribosomes, we propose that lysine acetylation is highly prevalent and dynamically regulated in ribosomes across the three kingdoms of life.

### The coordinated acetylation of r-proteins by Pat and AcP

Pat catalyze acetylation of various proteins enzymatically (20,23,49,65–67). Additionally, Weinert *et al.* demonstrated that AcP levels are correlated with acetylation levels *in vivo*, indicating that AcP could non-enzymatically acetylate proteins in bacteria (68). These two acetylation mechanisms may differ in some critical elements. It is possible that Pat performs enzymatic and acetyl-CoA-dependent acetylation while AcP-dependent acetylation could respond to time and/or glucose concentration (69). For instance, the important transcription regulatory protein PhoP can be acetylated by both Pat and AcP in *Salmonella*. Pat-mediated acetylation of PhoP at K201 inhibits its DNA binding ability, while AcP-mediated acetylation of K102 blocks PhoP phosphorylation (19,49). In this study, we isolated r-proteins from different stains cultured in LB medium and found that 200 Kac sites of r-proteins were regulated by both Pat and AcP. Since most bacterial genomes contain Pat homologs and the AcP metabolism pathway, the dual-pathway acetylation of r-proteins is highly likely to be conserved in bacteria.

### CobB is involved in regulating r-proteins acetylation

Reverse reaction of acetylation relies on deacetylases, including sirtuin deacetylases and histone deacetylases (HDAC) in eukaryotes (70). CobB, a sirtuin deacetylase isoform, can remove acetyl groups from Kac sites in the presence of NAD^+^ in bacteria (21,22). *S*. Typhimurium has a single CobB, while *Mycolicibacterium smegmatis* has one SIRT5 ortholog and one SIRT4 ortholog (71). Deletion of *cobB* can globally elevate protein acetylation level in *E. coli* (31). In this work, our mass spectrometry data showed that deletion of *cobB* increased the acetylation levels of only 95 lysine residues in r-proteins. There are some possible explanations for this. One is due to the high acetylation background maintained by Pat and intracellular AcP, which are mighty to acetylate lysine residues. Alternatively, CobB expression or activity might be regulated in some way. It is found that the secondary messenger c-di-GMP strongly binds to CobB and inhibits CobB activity (72). Since the level of c-di-GMP varies in response to environmental cues, we propose that CobB might be active only under certain scenarios.

Interestingly, we identified 13 Kac sites in L2, and the acetylation levels of these 13 lysine residues increased in *cobB* deletion mutant. Moreover, *in vitro* assays showed that L2 could be deacetylated by CobB, indicating that CobB indeed plays an important role in regulating L2 acetylation. These findings suggest that CobB can regulate ribosome function such as assembly and translation by deacetylating some specific r-proteins.

Chen *et al.* defined all of the ‘‘early group’’ primary binding proteins in the process of large subunit assemble by *in vitro* chemical probing experiments (73). Williamson *et al.* then reordered the assembly map using a combination of quantitative mass spectrometry and cryo-EM (74). They found that L3, L4, L20, L23 and L24 are likely the first bound to 23S rRNA strongly. In our work, we identified 9 Kac sites in L3, 10 in L4, 4 in L20, 6 in L23 and 6 in L24 totally. Among these Kac sites, only a few can be enzymatically reversed by CobB (4 out of 9 in L3, 3 out of 10 in L4, 0 out of 4 in L20, 2 out of 6 in L23 and 2 out of 6 in L24). So, this finding may explain why deletion of *cobB* did not disturb the ribosome assembly.

In the translation elongation cycle, aminoacylated tRNA is brought into the A site as a complex with EF-Tu and GTP which also need L7/L12 stalk (75). Our mass results identified 8 acetylated residues in L7/L12, of which Pat affected six residues, AcP affected 7 residues, and CobB deacetylated four residues. This can explain the similar translation efficiency defect we observed in Δ*pat*, Δ*cobB* and Δ*ackA*.

### Acetylation homeostasis is crucial for ribosome assembly and translation

Our mass spectrometry data revealed a significant increase in lysine acetylation of r-proteins during stationary phase compared to exponential phase. Specifically, 182 lysine sites in r-proteins exhibited elevated acetylation levels in stationary phase. Considering the differential expression of *pat* and *cobB* across growth phases and the accumulation of intracellular AcP in stationary phase (69), we propose that the coordinated action of these three acetylation-modulating factors are crucial for maintaining physiological ribosome acetylation levels. Polysome profiling showed that an appropriate level of r-protein acetylation is essential for facilitating functional 70S ribosome assembly, by ensuring proper association between rRNA and r-proteins. Notably, deletion of *pat* or *cobB* led to a slight impairment in 70S ribosome assembly and a dramatic attenuation in the binding of elongation factors EF-Tu, EF-Ts and EF-G to polysomes. Furthermore, AHA labelling assays demonstrated that disruption of ribosome acetylation status could hinder ribosome translation efficiency. Acetylation homeostasis was studied in the context of chromatin maintenance and gene expression in eukaryotes, which was influenced by the dose and enzymatic activity of histone acetyltransferases (HATs) and HDACs. Several studies have illustrated that balanced histone acetylation status within the nucleus is critical for neuronal vitality (76,77). Also, homeostatic status of histone acetylation is essential for auxin signaling as well as root morphogenesis in plants (78,79).

The ribosomal protein L24 is an assembly-initiator protein essential for early ribosome assembly (80), whereas spontaneously mutated L24 showed temperature-sensitive phenotype (81). Our mass spectrometry data revealed that L24 K33 could be acetylated by AcP, and crystal analysis showed L24 could associate with 23S rRNA (82). Unfortunately, our attempts to construct chromosomal mutation of L24 K33 were unsuccessful due to the essentiality of the protein (83). However, we confirmed that L24 could be acetylated by AcP and the acetylation homeostasis of K33 is crucial for rRNA binding *in vitro*. These findings suggest that AcP-mediated acetylation might regulate early ribosome assembly through r-protein and rRNA association.

Ribosomal-stalk protein L7/L12 (L7 is the N-terminal acetylated form of L12) is a two-domain protein composed of an N-terminal dimerization module and a globular CTD connected by a flexible hinge that allows it to acquire multiple conformations on the ribosome. L7/L12 binds as two dimers via their N-terminal domains (NTD) to L10, and L10 is attached to the rRNA. The CTD domain of L7/L12 is required for recruitment of elongation factors EF-Tu and EF-G to the ribosome and stimulates GTP hydrolysis by the ribosome bound factors through stabilization of their active GTPase conformation (5). Diaconu *et al.* previously showed that the mutation of L7/L12 K65 reduced the binding of EF-Tu and GTP hydrolysis (4). Our data showed that L7/L12 K65 and K70 could be acetylated by both Pat and AcP, and (de)acetylation-mimic mutations of K65Q, K65R, K70Q and K70R reduced the ribosome assembly and EF-Tu-binding entirely. These findings suggest that appropriate acetylation status of K65 and K70 is required for rRNA and EF-Tu recruitment and subsequent assembly and translation elongation efficiency.

Recently, Feid *et al.* report that acetylation of r-proteins inhibits the formation of 70S ribosomes and impairs protein translation in *E. coli* (63). There are some commonalities between our results. Regarding the ribosome dissociation pattern, they also observed that both the Δ*ackA* strain (high-acetylation) and the Δ*pta* strain (low-acetylation) had smaller 70S peak than the WT strain. This indicates that mutants with different acetylation states had defect in ribosome assembly. Moreover, they showed that the polysome profiles of the Δ*ackA* diverged from that of the WT at later time points, as cells exited exponential phase. Feid *et al.* argue that translation regulation by acetylation is a dynamic phenomenon that is growth-phase specific. In line with their results, we also conclude that the r-proteins undergo dynamic acetylation in bacterial cells. Our data further extend that acetylation homeostasis is important for bacterial translation processes.

**Figure 7.**
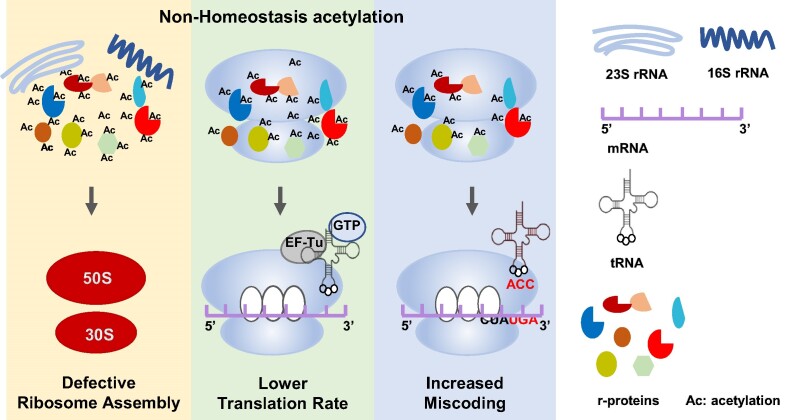
Working model demonstrating the role of acetylation homeostasis in ribosome assembly and function. Normal r-proteins possess acetylation homeostasis, which is crucial for ribosome assembly and protein translation efficiency and fidelity. Hyper- or hypo-acetylation of ribosomes (non-homeostasis) may lead to defective ribosome assembly, low translation efficiency and high miscoding rate.

Therefore, we propose a model (Figure 7) to decipher how bacteria utilize protein acetylation to mediate ribosome assembly, translation efficiency and fidelity, which represents a novel form of environmental adaptation in bacteria.

## Supplementary Material

gkad768_Supplemental_Files

## Data Availability

The data underlying this article are available in the article and in its online supplementary material.
