# Supplementary material for: Global profiling of ribosomal protein acetylation reveals essentiality of acetylation homeostasis in maintaining ribosome assembly and function
Source: Nucleic Acids Res. 2023 Sep 23;51(19):10411–27. doi: 10.1093/nar/gkad768 (PMC10602876; doi:10.1093/nar/gkad768)

Supplementary figure legends

Fig. S1. Lysine acetylation is widely present in ribosome proteins.

(A) Workflow for LC-MS/MS based analysis of ribosome proteins acetylation. The ribosomes from wild-type *S.* Typhimurium strain 14028S (WT), Δ*pat*, Δ*cobB* and Δ*ackA* in log phase and WT in stationary phase were collected using sucrose gradient centrifugation. The acetylated peptides were enriched by anti-Kac antibody beads and followed by LC-MS/MS analysis.

(B) Analysis of the percentage of lysine residues in non-ribosome and ribosome proteins in *S.* Typhimurium strain 14028S.

(C) The correlation analysis of acetylated lysine residues and total number of lysine residues in *Salmonella* r-proteins*.*

Fig. S2. Acetylation alters ribosome association with translation factors, translation efficiency and fidelity.

(A) Clusters of Orthologous Genes pathway analysis of polysome-binding proteins decreased in Δ*ackA* compared to WT.

(B) Analysis of acetylation levels of EF-Tu, EF-Ts and EF-G in Δ*ackA* by LC-MS/MS.

Fig. S3. Acetylation of L24 K33 is involved in ribosome assembly.

(A) The structure of L24 and 23S rRNA (PDB number: 4V6G). The analysis of L24 K33 involved in the interactions between L24 (indicated in blue) and 23S rRNA (indicated in shadow) was performed using the PyMol software.

(B) The His-tagged L24, L24 K33Q, K33R, and K33A proteins were purified by Ni-NTA column. The homogeneity of these proteins was determined by SDS-PAGE.

Fig. S4. Acetylation of L7/L12 K65 and K70 is involved in ribosome assembly and association to EF-Tu, translation efficiency and fidelity.

(A) Alignment of L7/L12 amino acid sequence from various bacterial species including *Salmonella enterica*, *Escherichia coli*, *Thermus thermophilus*, *Mycobacterium tuberculosis*, *Haemophilus influenzae*, *Bordetella avium*, *Bacillus subtilis*, *Streptococcus pneumoniae*. Asterisks indicate the conserve lysine residues.

(B) The comparison of acetylation level of K65, K70, K81 and K84 in L7/L12 in mutant strains and different growth phases by LC-MS/MS (Raw data are from supplementary Table 3).

(C) The structure of EF-Tu and L7/L12 (PDB number:1EFU, PDB number: 1CTF). The analysis of L7/L12 K65 and L7/L12 K70 involved in the interactions between L7/L12 (indicated in blue) and EF-Tu (indicated in yellow) was performed using the PyMol software.

Supplementary tables

Table S1 Bacterial strains and plasmids used in this study.

Table S2 Primers used in this study.

Table S3 Quantitative analysis of ribosome proteins acetylation by LC-MS/MS.

Ribosomes from WT, Δ*pat*, Δ*cobB*, and Δ*ackA* were isolated by sucrose gradient centrifugation and applied to LC-MS/MS analysis after anti-Kac antibody enrichment. The acetylation levels of all acetylated r-protein residues were quantified individually.

Table S4 Quantitative analysis of polysomes by LC-MS/MS.

Polysomes from WT and Δ*ackA* were isolated by sucrose gradient centrifugation and applied to LC-MS/MS analysis. The levels of all r-protein and ribosome associated

proteins were quantified and listed in sheet 1. Decreased ribosome associated proteins in Δ*ackA* were listed in sheet 2.

Figure S1

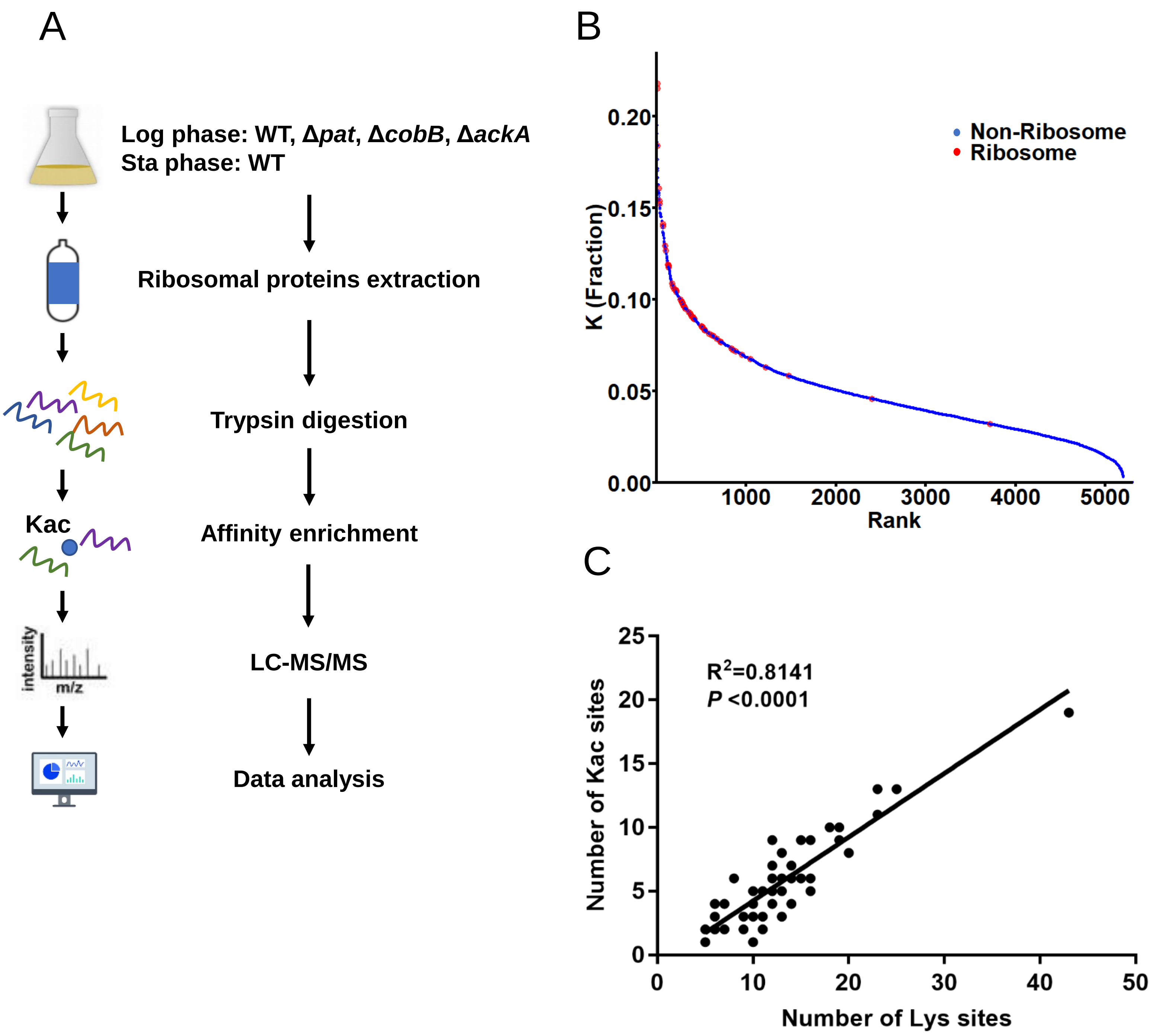

A
Log phase: WT, Δpat, ΔcobB, ΔackA
Sta phase: WT
Ribosomal proteins extraction
Trypsin digestion
Kac
Affinity enrichment
intensity
m/z
LC-MS/MS
Data analysis
B
K (Fraction)
0.20
0.15
0.10
0.05
0.00
1000
2000
3000
4000
5000
Rank
Non-Ribosome
Ribosome
C
Number of Kac sites
25
20
15
10
5
0
0
10
20
30
40
50
Number of Lys sites
R2=0.8141
P <0.0001

Figure S2

A

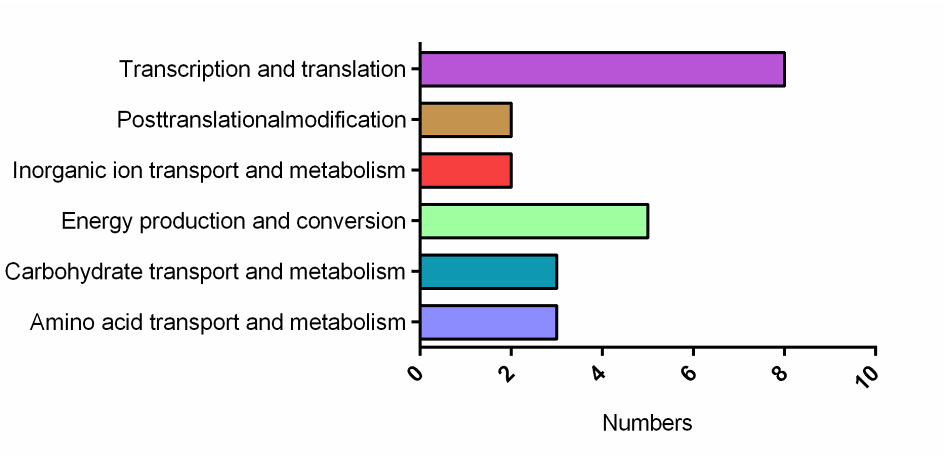

B

Ribosome associated proteins（RAPs）	*ΔackA*/WT (fold change)
**EF-Tu**	0.62
**EF-Ts**	0.64
**EF-G**	0.58

Figure S3

A

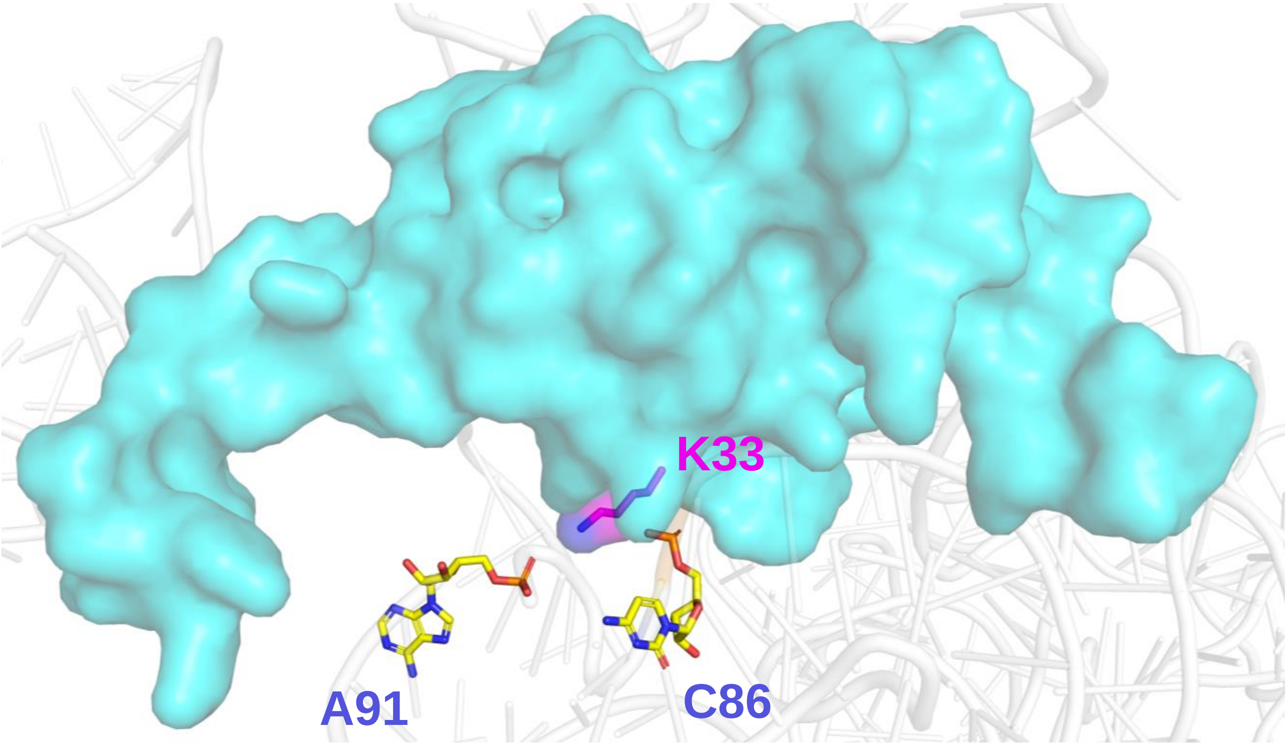

B

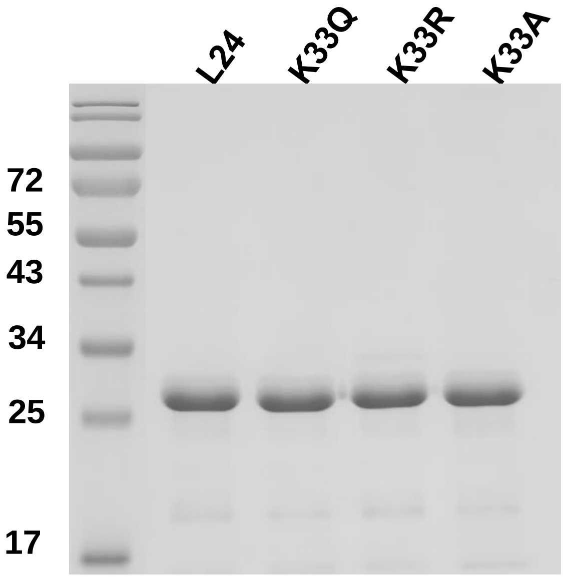

Figure S4

A

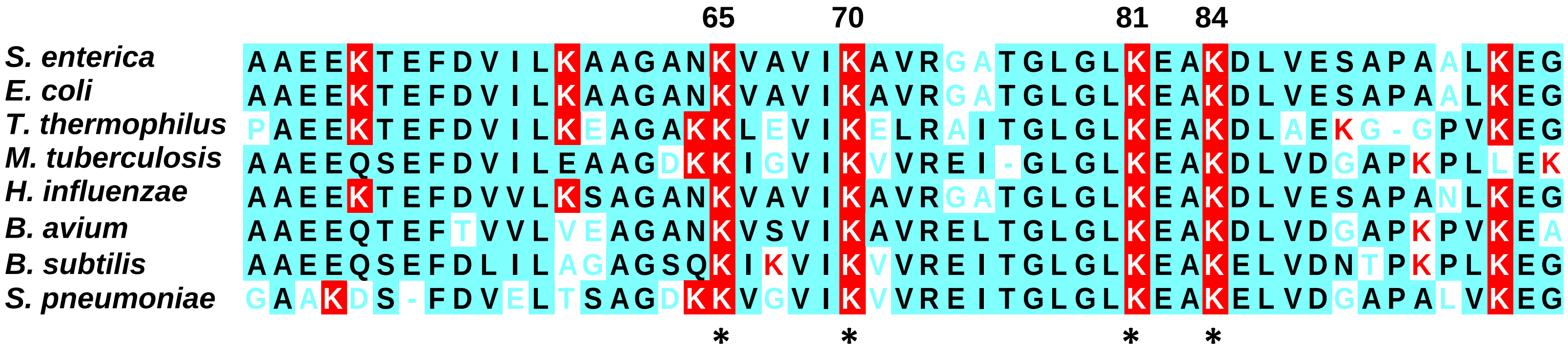

B

L7/L12	Δ*pat*/WT (fold change)	Δ*ackA*/WT (fold change)	WT sta/WT log (fold change)
K65	0.15	11.40	2.99
K70	0.12	6.19	5.78
K81	0.11	9.12	0.49
K84	0.26	0.62	1.30

C

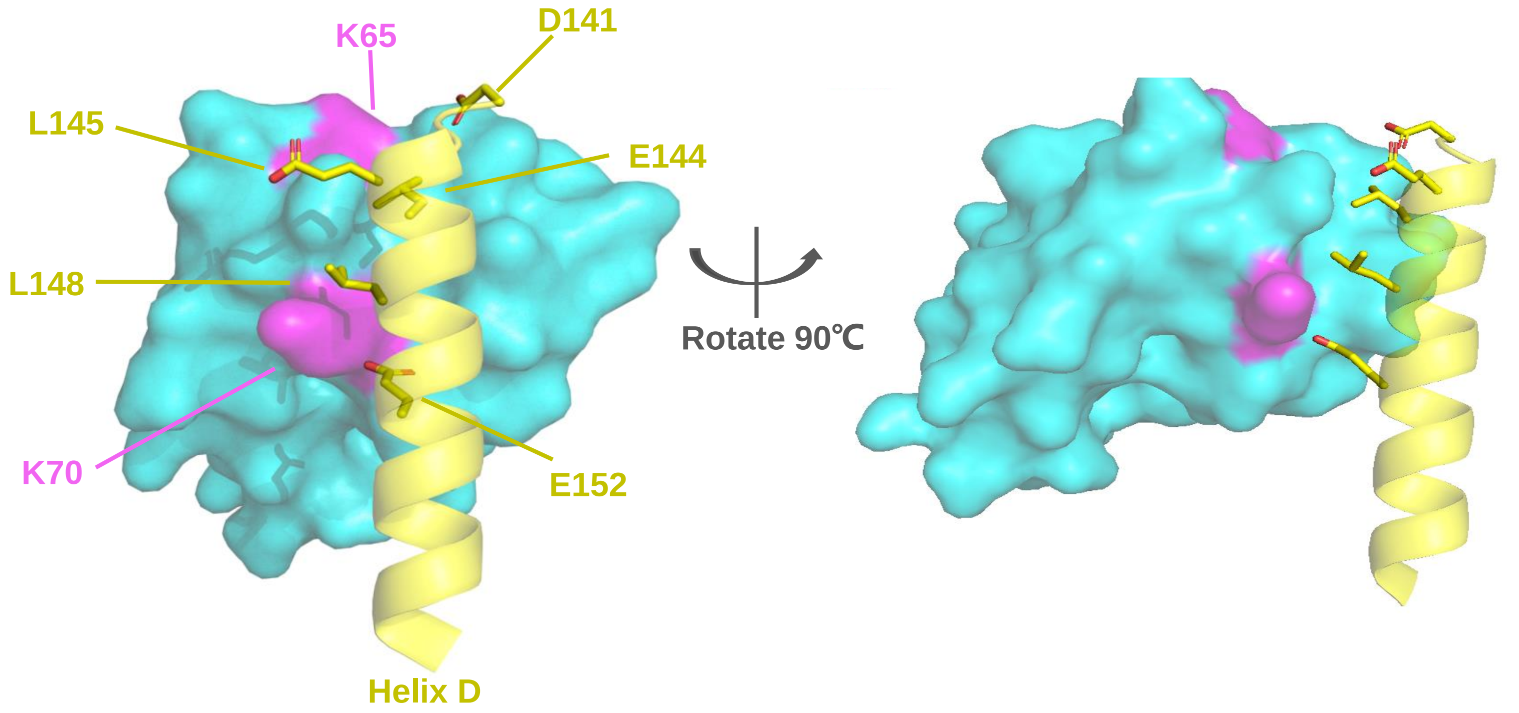

Supplement: gkad768_Supplemental_Files [file gkad768_supplemental_files.zip › supplementary figure and legend-20230812.pdf]
